# Hydroelectricity Generation from Fiber-Oriented Waste Paper via Capillary-Driven Charge Separation

**DOI:** 10.3390/polym17212945

**Published:** 2025-11-04

**Authors:** Hyun-Woo Lee, Seung-Hwan Lee, So Hyun Baek, Yongbum Kwon, Mi Hye Lee, Kanghyuk Lee, Inhee Cho, Bum Sung Kim, Haejin Hwang, Da-Woon Jeong

**Affiliations:** 1 Korea National Institute of Rare Metals, Korea Institute of Industrial Technology, Incheon 21655, Republic of Korea; totptkd12@kitech.re.kr (H.-W.L.);; 2 Department of Material Science Engineering, Inha University, Incheon 22212, Republic of Korea; 3 School of Mechanical Engineering, Chung-Ang University, Seoul 06974, Republic of Korea

**Keywords:** sustainable energy, waste-to-energy, hydroelectricity, wasted printing paper, upcycled materials

## Abstract

Hydroelectricity energy harvesting has emerged as a promising, eco-friendly alternative for addressing the growing demand for sustainable energy solutions. In this study, we present a hydroelectricity energy harvester fabricated from shredded waste printing paper (WPP), offering a novel waste-to-energy conversion strategy that requires neither material purification nor complex processing. The device leverages the randomly entangled fiber network of WPP to facilitate capillary-driven moisture diffusion and electric double layer (EDL) formation, thereby enabling efficient electrokinetic energy conversion. The random arrangement of WPP fibers increases the effective EDL area, allowing the waste printing paper generator (WPPG) to achieve an open-circuit voltage of 0.372 V and a short-circuit current of 135 μA at room temperature under optimized electrolyte conditions. This study demonstrates that carbon-black-coated WPP can be effectively upcycled into a high-performance hydroelectricity generator, exhibiting excellent electrical output at ambient conditions. By combining material recycling with efficient energy conversion, this system establishes a practical and sustainable pathway for distributed power generation. Overall, this work not only presents an environmentally responsible approach to device fabrication but also highlights that hydroelectricity energy harvesting using WPPG represents a promising alternative energy route for future applications.

## 1. Introduction

The energy demand of modern economy and society is continuously increasing, stimulating ongoing and growing interest in the development of sustainable energy resources. Unfortunately, most current energy generation and conversion systems still rely heavily on fossil fuels, giving rise to significant environmental problems [1,2,3]. The combustion of fossil fuels emits harmful substances such as carbon dioxide, fine dust, and nitrogen oxides, which are major causes of air pollution and climate change and pose a severe threat to human health and the global ecosystem [4,5,6,7,8]. Additionally, fossil fuel resources are finite, and their continued use undermines long-term energy security. Therefore, it is urgently necessary to develop alternative, sustainable, and eco-friendly energy technologies [9,10].

Small-scale energy harvesting is one such technology that enables the collection of low-level environmental energy and its conversion into usable electricity. This approach is gaining attention due to its potential for application in powering autonomous sensors or portable electronic devices without relying on existing power grids. Various mechanisms for small-scale energy harvesting have been proposed, including piezoelectric, triboelectric, thermoelectric, and evaporation-based systems [11,12,13,14,15,16,17,18].

Evaporation-based energy harvesting generates electricity by utilizing capillary flow and moisture-induced stress gradients created by the natural evaporation of water. This method provides a continuous and self-sustaining power output at room temperature with minimal structural complexity and environmental impact. The performance of hydroelectricity generators depends heavily on the physical and chemical properties of the energy-harvesting materials [19,20].

Previous research on evaporation-based energy harvesting has primarily focused on enhancing porosity, hydrophilicity, and conductivity of the harvesting materials using synthetic substrates such as metal oxides, cellulose acetate, and carbon nanomaterials. In particular, cellulose-based materials have attracted significant attention due to their abundance, biodegradability, and natural porous structure [21,22,23,24,25,26]. However, the use of these materials often incurs high costs, involves complex manufacturing processes, and provides limited environmental benefits. In contrast to the studies of cellulose-based materials, research on the utilization of waste resources as functional substrates for evaporation-based energy harvesting systems has been scarce.

Despite the widespread adoption of digital media, paper consumption remains high across industrial and educational sectors, resulting in significant waste generation. In the United States, approximately 110 million tons of waste paper are generated annually, while in Europe, approximately 11 million tons of pulp and paper waste are produced annually [27,28]. Most of this waste is incinerated or landfilled, contributing to deforestation, greenhouse gas emissions, and land scarcity [29,30,31,32,33,34].

This study presents the first evaporation-based energy-harvesting device using recycled wasted printing paper (WPP), which we name the WPP generator (WPPG). By coating discarded paper with a conductive carbon layer, we have developed a simple, inexpensive, and environmentally friendly system that can generate electricity from ambient moisture. Furthermore, this study systematically compared two fabrication strategies producing WPPG-stack and WPPG-shred devices to identify the optimal structural configuration for maximizing device performance. The results showed that WPPG-shred exhibits superior performance compared to WPPG-stack, because the irregular entanglement of fibers in WPPG-shred forms more microchannels and complex channels, increasing the effective interface area for electrical double layer (EDL) formation. WPPG-shred exhibited excellent performance, achieving a maximum open-circuit voltage of 0.372 V and a short-circuit current of 135 μA at room temperature.

## 2. Experimental Section

### 2.1. Fabrication of CB Solution and WPPG

**Figure 1:**
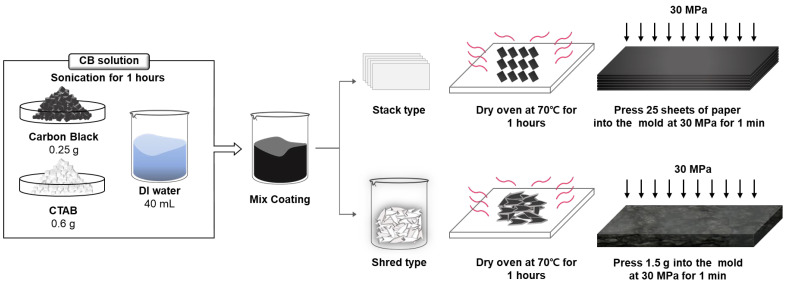
Schematic diagram of the CB coating solution and the process for the fabrication of WPPG-stack and WPPG-shred.

The fabrication of WPPG-stack and WPPG-shred is schematically illustrated in Figure 1. For WPPG fabrication, CB powder (Ketjen Black EC 600 JD, Lion Co., Ltd., Tokyo, Japan) and cetyltrimethylammonium ammonium bromide (CTAB, Tokyo Chemical Industry Co., Ltd., Tokyo, Japan) at the concentrations of 6.25 g/mL and 15 g/mL, respectively, were dispersed in deionized water (40 mL) to prepare a conductive coating solution. After brief stirring, the mixture was subjected to ultrasonic treatment for 1 h. CTAB acts as a surfactant, adsorbing onto the carbon surface to increase the electrostatic repulsion and enhance colloidal stability, thereby stabilizing the dispersion [35]. This process ensures a uniform distribution of CB particles and prevents particle agglomeration during storage and use.

For WPPG-shred, WPP was shredded into small arrow-shaped pieces with the dimensions of approximately 1 mm × 4 mm. The shredded paper (20 g) was mixed with the prepared CB solution to ensure that the fibers were completely impregnated. The impregnated mixture was then placed in a drying oven and dehydrated at 70 °C for 1 h to remove residual moisture and fix the conductive coating on the paper surface.

After drying, the coated paper (approximately 1.5 g) was molded into a rectangular shape with the dimensions of 20 mm × 40 mm using a stainless-steel mold. The sample was compressed at 30 MPa for 1 min using a hydraulic press to produce a dense, self-supporting membrane, which was named WPPG-shred. The final device had a thickness of approximately 1.9 mm and exhibited sufficient structural stability for further testing.

For WPPG-stack, WPP sheets were first cut into uniform rectangles with the dimensions of 20 mm × 40 mm. Each sheet was coated with the same CB solution to ensure consistent conductivity across the entire surface. After drying, 25 sheets were stacked and compressed at 30 MPa for 1 min within the same stainless-steel mold, resulting in a final thickness of approximately 1.8 mm. This structure was named WPPG-stack.

While both types of devices share the same coating and compression conditions, they have fundamentally different internal structures. WPPG-stack has a layered structure, while the structure of WPPG-shred is formed by randomly intertwined shredded fibers. For electrical property measurements, electrodes were attached to both ends of the WPPG, and a calcium chloride solution (300 μL, ≥99.98%, Sigma-Aldrich Co., Ltd., St. Louis, MO, USA) was injected into one end to begin performance evaluation.

### 2.2. Electrical Measurements and Data Analysis for WPPG

The morphology of WPPG was observed using a field emission scanning electron microscope (FE-SEM, JSM-7100F, JEOL Ltd., Tokyo, Japan). The functional groups on the WPPG surface were analyzed by Fourier transform infrared spectroscopy (FT-IR, VERTEX 80V, Bruker, Billerica, MA, USA), and the resistance was evaluated using a digital multimeter (Fluke 17b+, Fluke Corporation, Everett, WA, USA). All experiments were conducted in an acrylic chamber with controlled temperature and humidity that was equipped with a humidifier and dehumidifier (Figure S1). The environmental conditions were continuously monitored using an Arduino Uno system (SZH-EK002, Arduino LLC, Somerville, MA, USA). The open-circuit voltage (*V*_oc_) and short-circuit current (*I*_sc_) were measured using a precision source meter (Keithley 2400, Keithley Instruments, Cleveland, OH, USA), and device control and data collection were performed using IV Solution software 2400S (I.V. Solution, Seoul, Republic of Korea). *V*_oc_ and *I*_sc_ were recorded for 1000 s, and the average value within the stable interval of 600–900 s was calculated to ensure stability and reliability. Four repeated measurements were performed under the same conditions to ensure statistical consistency.

## 3. Results and Discussion

### 3.1. WPPG Characterization

**Figure 2:**
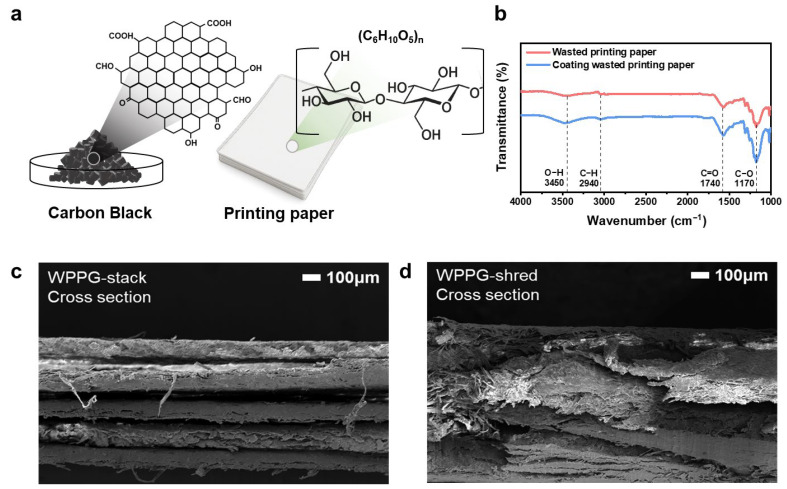
(**a**) Chemical structure of CB and WPP. (**b**) Fourier transform infrared spectra of WPP and coated WPP. Cross-sectional SEM image of (**c**) WPPG-stack and (**d**) WPPG-shred.

To evaluate the structural, chemical, and morphological changes caused by CB coating, various characterization analyses were performed on WPP prior to CB coating and on the fabricated hydroelectricity generator (WPPG), with the results summarized in Figure 2a. The CB-based composite coating used in this study was introduced to enhance the surface conductivity and hydrophilicity of WPP. As schematically shown in Figure 2a, the CB used in this study was not the original powder but rather was functionalized with hydrophilic groups, primarily hydroxyl (-OH) functional groups. These polar functional groups enhance the wettability of the CB surface, facilitating moisture absorption and transport. CB retains its inherent electrical conductivity, and, as observed in the TEM image in Figure S6, the CB particles have sizes below 50 nm, which contribute to their high specific surface area, making CB a conductive material capable of simultaneously transporting moisture and charge [36,37].

The WPP substrate is primarily composed of cellulose, which contains abundant hydroxyl groups along its polymer chains [38]. The structural compatibility between CB and cellulose facilitates uniform coating during the manufacturing process. The resulting hybrid material offers the advantages of enhanced ion mobility due to the hydrophilic functional groups of the functionalized CB surface and the improved charge transport through the conductive CB network.

FT-IR spectroscopy was used to analyze the surface chemistry of WPP before and after CB coating (Figure 2b). Pure WPP exhibited the characteristic peaks of cellulose, including O–H stretching at 3450 cm^−1^, C–H stretching at 2940 cm^−1^, C=O stretching at 1740 cm^−1^, and C–O stretching at 1170 cm^−1^. After CB coating, the O–H and C–O bands were slightly broadened and their intensities increased, indicating surface modification and the presence of carbon-based materials.

Field emission scanning electron microscopy (FE-SEM) analysis (Figure S2) confirmed that WPPG exhibits a densely packed microstructure in which cellulose fibers and CB particles are intertwined. CB particles were uniformly coated on the fiber surface and partially embedded within the cellulose matrix, forming a continuous and conductive network. This dense structure enhances interfacial contact and facilitates moisture-based charge transport, which is essential for hydroelectricity power generation. The inherent pore structure of the cellulose substrate provides effective moisture diffusion pathways and supports continuous moisture absorption throughout the device. This incorporation of CB not only enhances mechanical stability but also establishes a continuous conductive pathway throughout the material. The CB coating is well-integrated and does not block the pore structure of the paper; thus, it plays an essential role in maintaining moisture interaction in generator applications.

To further confirm the surface composition and successful CB coating, EDS elemental mapping was performed, with the results shown in Figure S2. The mapping results showed widely distributed signals of carbon (C) and oxygen (O) across the surface; however, since both the cellulose of WPP and CB contain these elements, the presence of C and O cannot be used to distinguish between WPP and CB. To address this limitation, bromine (Br) and nitrogen (N) from cetyltrimethylammonium bromide (CTAB), used in the CB coating solution, were introduced as tracer indicators. The uniform and overlapping distributions of Br and N in the elemental mapping provide an indirect confirmation of the successful application of the CB coating on the WPP surface. These results demonstrate that CB assisted by CTAB is well-dispersed throughout the fiber network, enabling moisture-based energy harvesting.

The cross-sectional structure of the fabricated WPPG was observed by FE-SEM, and the results are presented in Figure 2c,d. For WPPG-stack (Figure 2c), it was observed that the paper sheets were uniformly and regularly stacked to form a layered structure. By contrast, WPPG-shred (Figure 2d) showed an entangled structure formed by randomly arranged shredded paper pieces with non-uniform shapes and complex pathways. The fibers are irregularly arranged to form fluid paths in various directions, exhibiting more complex structural characteristics.

### 3.2. Optimization of WPPG Performances Through Control of Internal

**Figure 3:**
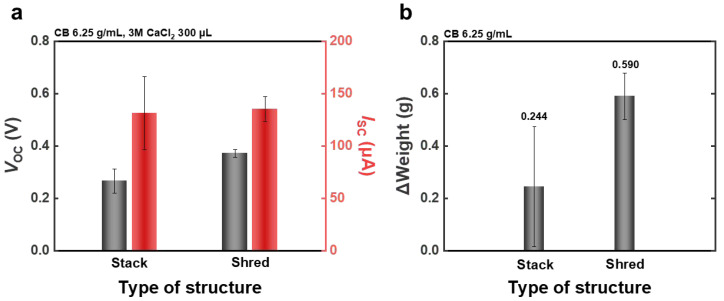
(**a**) *V*_oc_ and *I*_sc_ for the two WPPG structures. (**b**) Average weight change in WPPG after immersion in deionized water for 5 s.

To evaluate the effect of structural configuration on device performance, two types of hydroelectricity generators were compared under identical conditions: WPPG-stack and WPPG-shred. All devices were fabricated using WPP and tested using a 3M CaCl_2_ solution (300 μL). The physical and structural characteristics of the devices are summarized in Table S1. The raw data for Figure 3a is provided in Figure S3.

The WPPG-stack device was fabricated by compressing 25 sheets of coated paper (20 mm × 40 mm) into a stacked structure with a final thickness of 1.8 mm. As shown in Figure 3a, the WPPG-stack exhibited an average *I*_sc_ of 131 μA and a voltage of approximately 0.266 V. By contrast, the WPPG-shred, fabricated using shredded waste paper fragments (approximately 1 mm × 4 mm), demonstrated superior performance with an *I*_sc_ of 135 μA and a *V*_oc_ of 0.372 V, as also presented in Figure 3a. The average and raw data for the optimization of the WPPG-shred under generator width and length, injected solution types, and solution volumes are presented in Figures S11–S16. While the *I*_sc_ of the WPPG-shred is comparable to that of the WPPG-stack, its *V*_oc_ is approximately 0.1 V higher.

Although the two devices have similar thicknesses and weights, their internal structures and fiber arrangements differ significantly, leading to the observed differences in *V*_oc_. WPPG-stack features a laminated, stacked architecture. During hydroelectricity power generation, the introduction of water at one end led to localized wetting, and the formation of continuous fluid pathways was limited. The limited internal volume restricted ion mobility and fluid flow, thereby reducing the dynamic formation and evolution of the EDL, which is crucial for charge transport and storage efficiency of the device [39]. Consequently, the effective solid–liquid interfacial area available for EDL formation was relatively small [40,41].

In contrast to WPPG-stack, WPPG-shred is composed of densely packed entangled paper pieces, forming a highly porous structure with high pore connectivity unique to randomly entangled fiber structures [35,42]. The irregular entanglement of these fibers naturally forms numerous microchannels and intricately connected channels, effectively promoting moisture penetration and diffusion. Since the WPPG-shred structure provides wider channels for faster moisture transport, the effective interface area for EDL formation across the electrode increases even under the same amount of moisture [42]. Accordingly, WPPG-shred has more pore channels than WPPG-stack, which is advantageous for capillary flow and ion diffusion, and achieves a larger potential difference, resulting in superior electrical performance [35].

The structural differences between the two WPPG materials were further evaluated and verified through water absorption tests. In this experiment, each WPPG device was placed vertically on a Petri dish containing deionized water (10 mL) for 5 s to induce water absorption, and the weight change before and after absorption was measured. This test was repeated five times for each device type, and the average values calculated excluding the highest and lowest measured values are presented in Figure 3b. The detailed numerical data are presented in Table S2.

It was found that WPPG-shred absorbed an average of 0.590 g of water, while WPPG-stack achieved a relatively small water absorption amount of 0.244 g. This result is consistent with previous reports that WPPG-shred has high porosity and flow connectivity due to its randomly entangled fiber structure which facilitates water penetration and enables a absorption of a large amount of water. This difference reflects the effect of various fluid channels within the fiber structure that rapidly and widely distribute water, which is also consistent with the water absorption behavior shown in Figure 3b [35,42].

Therefore, this difference in the water absorption behavior is due to the structural characteristics of WPPG-shred, which not only promote fluid transport through capillary flow, but also play an important role in the formation and maintenance of the EDL by improving the uniformity of the water distribution within the electrode. Thus, it can be confirmed that WPPG-shred has a key structural advantage that endows it with superior electrical output characteristics compared to WPPG-stack [35,42].

### 3.3. Optimization of WPPG-Shred Performance Through Control of CB Concentration

**Figure 4:**
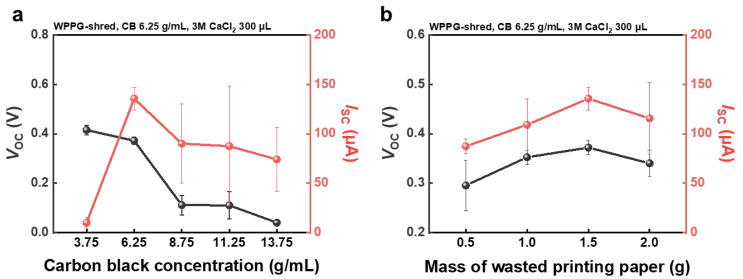
(**a**) Dependence of *V*_oc_ and *I*_sc_ of WPPG-shred on CB concentration. (**b**) Dependence of *V*_oc_ and *I*_sc_ of WPPG-shred on WPP mass.

Building upon the superior performance of WPPG-shred, further investigations were conducted to examine how the conductivity of WPPG-shred influences its electrical output. Specifically, the effect of CB concentration on hydroelectricity generation was systematically evaluated while maintaining the same structural parameters. The performance as a function of carbon black concentration is shown in Figure 4a, and the corresponding raw data are provided in Figure S4.

To investigate the influence of CB concentration on hydroelectricity generation, WPPG devices were fabricated with identical structural parameters: a planar area of 20 × 40 mm, a total mass of 1.5 g, and a thickness of 1.9 mm. Here, only the CB content in the coating solution was varied, ranging from 3.75 g/mL to 13.75 g/mL, while all other fabrication conditions were kept constant to isolate the effects of carbon loading on device performance.

As shown in Figure S5, the zeta potential of the coating solution increased with CB concentration, exceeding 6.25 g/mL and plateauing near 30 mV, indicating the formation of a colloidally stable dispersion. This electrostatic stability prevents the aggregation of CB particles and promotes uniform coating distribution within the cellulose fiber network of WPPG after coating. This uniformity enhances water interactions and EDL formation, which are essential for streaming current generation.

The trend of the changes in the zeta potential with respect to CB concentration closely mirrors the behavior of *I*_sc_, which rises steeply up to a CB concentration of 6.25 g/mL before decreasing, demonstrating the importance of interfacial surface charge in hydroelectricity generation. The electrical output characteristics of the WPPG devices are summarized in Figure 4a. The device fabricated with 6.25 g/mL CB exhibited optimal performance, achieving a *V*_oc_ of 0.372 V and an *I*_sc_ of 135 μA. By contrast, the sample with 3.75 g/mL CB showed a slightly higher *V*_oc_ of 0.415 V but a significantly lower *I*_sc_ of 10.2 μA, reflecting a more than 13-fold enhancement in the short-circuit current upon CB optimization.

Additionally, the resistance decreased significantly from 62.7 kΩ to 6.25 kΩ, suggesting the formation of continuous conductive pathways throughout the device (Figure S7). However, increasing the CB content beyond 6.25 g/mL resulted in a gradual decline in both *V*_oc_ and *I*_sc_, despite the continuing reduction in the resistance. This performance degradation is attributed to excessive CB accumulation, which may obstruct the pores of the cellulose substrate and impede moisture diffusion.

As a result, the electrical conductivity increases with increasing CB concentration, resulting in a lower resistance, but the fluid flow pathways are partially blocked due to supersaturation of CB, resulting in a decrease in *V*_oc_ and *I*_sc_. These results suggest the importance of the appropriate concentration of CB in hydroelectricity power generator systems.

### 3.4. Optimization of WPPG-Shred Performance Through Control of WPPG-Shred Mass

To investigate the effect of mass changes in WPPG-shred on the performance, devices with different masses of 0.5, 1.0, 1.5, and 2.0 g were fabricated. The raw data of *V*_oc_ and *I*_sc_ are presented in Figure S8. All samples maintained the same planar size (20 mm × 40 mm) and CB concentration (6.25 g/mL). In addition, the measured resistance values of the devices are shown in Figure S9.

As shown in Figure 4b, the electrical performance improved as the WPP mass increased up to 1.5 g and then decreased at 2.0 g. WPPG-shred with the mass of 1.5 g showed the highest energy harvest output, with a *V*_oc_ of 0.372 V and *I*_sc_ of 135 μA. By contrast, the *V*_oc_ and *I*_sc_ values of the 0.5 g device were only 0.296 V and 87.3 μA, respectively, and the device also showed a significantly higher resistance of 14.7 kΩ compared to 6.21 kΩ for the device with the mass of 1.5 g. This represents an improvement of over 55% in open-circuit voltage and over 50% in the short-circuit current, suggesting that increasing substrate mass enhances charge generation and transport efficiency.

The performance improvement at intermediate mass values is attributed to the formation of a denser yet porous cellulose matrix, which results in increased material volume and the development of interconnected microchannel networks. These channels are advantageous for capillary-based water transport and EDL formation, both of which are essential for streaming current generation. Additionally, since the device is fabricated within a mold, an increase in the substrate mass leads to increased thickness, which reduces resistance due to improved ion retention and higher contact area.

However, the performance decreased when the substrate mass exceeded 1.5 g. Even though the lowest resistance (4.01 kΩ) was observed for the WPPG-shred with a mass of 2.5 g, both *V*_oc_ and *I*_sc_ decreased. This is attributed to the excessive densification of the cellulose network, which improved the conductivity but caused the previously open microchannels to collapse or shrink, thereby hindering moisture diffusion. As a result, EDL formation was limited, offsetting the favorable effect of the reduced resistance.

This structural trend is also supported by the measured thickness and density data presented in Figure S10. The thickness increased from 0.810 mm (0.5 g) to 2.46 mm (2.0 g), but the rate of increase gradually decreased, indicating that the cellulose layer was approaching its structural limit. The density also increased from 0.78 g/cm^3^ to 1.04 g/cm^3^, showing saturation between 1.5 g and 2.0 g (1.00 and 1.04 g/cm^3^, respectively). This increase indicates a transition from an open porous matrix to a dense structure, with a reduction in the effective pore volume that impedes water flow.

These results suggest that the structural stability and electrical performance of WPPG are determined by the substrate mass and the corresponding thickness adjustment. Therefore, optimization of the substrate mass and the corresponding porous structure is essential to ensure reliable device performance.

### 3.5. Comparison of Output Power of Cellulose and Paper-Based Energy Harvesters Reported in Recent Studies

**Figure 5:**
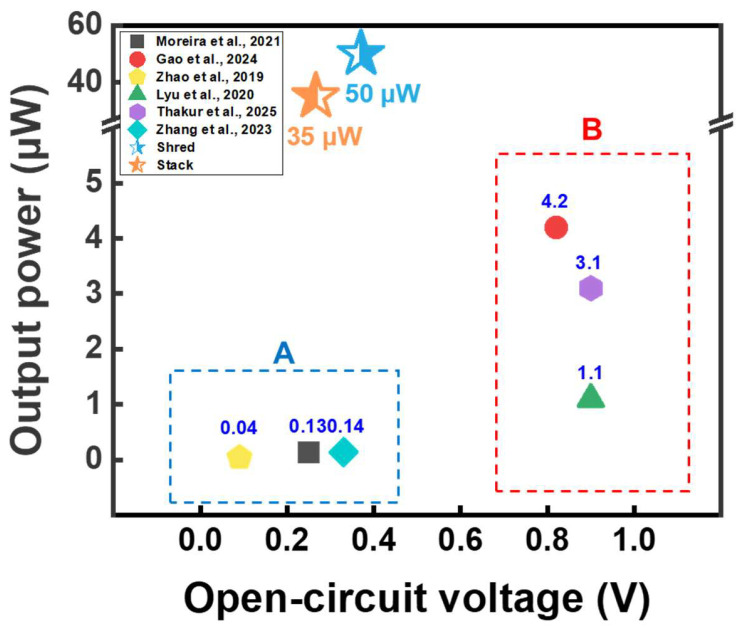
Comparative analysis of *V*_oc_ and output power for cellulose and paper-based hydroelectricity energy harvesting technologies reported by various studies [43,44,45,46,47,48]. (A) Recent hydroelectric energy harvesting studies with low output values. (B) Recent high-power hydroelectric energy harvesting studies.

As illustrated in Figure 5, WPPG outperforms many previously reported systems, despite being constructed entirely from post-consumer waste without using either refined cellulose or complex manufacturing processes [43,44,45,46,47,48]. The output power values in the region A in Figure 5 were only 0.13, 0.04, and 0.14 μW, respectively, indicating performance with limited suitability for practical use [43,45,48]. By contrast, region B in Figure 5 showed higher *V*_oc_ values but the corresponding current outputs remained low, resulting in modest power generation below 5 μW [44,46,47].

Most of the recently reported cellulose and paper-based energy harvesting devices have been manufactured in the form of thin sheets. Although these sheet structures have advantages such as flexibility, they suffer from limitations in the amount of moisture that can be absorbed and the evaporation area. Meanwhile, the WPPG used in this study was fabricated in the form of a 3D substrate based on WPP, which has structural advantages that enable greater moisture absorption and evaporation. In addition, by optimizing the internal structure, a randomly entangled fiber network was formed, which maximized the moisture transport and charge transfer path by capillary phenomenon, thereby improving the energy harvesting performance [35,42]. These structural characteristics played an important role in the optimization of the device. Owing to these characteristics, the obtained power output was higher than those of the existing paper-based energy harvesting devices. Unlike some reported devices that showed relatively high voltage but did not achieve sufficient current, which limited the actual output, WPPG achieved high energy output by generating relatively high current and voltage. Additionally, unlike many previously reported systems that can only operate effectively under specific humidity and temperature conditions, WPPG shows stable performance at ambient conditions. Furthermore, WPPG realizes energy conversion using waste materials processed using a simple fabrication procedure without necessitating complex purification procedures, which represents an important achievement that facilitates the practical implementation of this technology. Furthermore, considering the large amount of waste paper generated globally each year, a simple scaling analysis based on the annual waste paper output (approximately 110 million tons in the U.S.) indicates a theoretical power generation potential of about 3.67 GW. This value is a conceptual figure derived from simple calculations to illustrate the potential scale of energy conversion, rather than a realistic projection. It emphasizes the possibility of utilizing abundant waste resources as sustainable feedstocks for decentralized energy generation under ambient conditions.

## 4. Conclusions

In this study, a sustainable and high-performance energy harvesting device was developed using waste printing paper (WPP) as the sole substrate material. The WPPG-shred achieved a maximum open-circuit voltage (*V*_oc_) of 0.372 V and a short-circuit current (*I*_sc_) of 135 μA, resulting in a peak output power of 50 μW under ambient conditions. These results position the WPPG among the most efficient paper-based energy harvesting systems reported to date. Through comprehensive optimization of key device parameters, including substrate mass, planar dimensions, electrolyte concentration, internal structure, and interfacial configuration, the device effectively exploited the intrinsic advantages of its disordered fiber network. This unique architecture promotes directional capillary flow, enhances ionic mobility, and stabilizes electric double-layer formation, thereby enabling efficient moisture-to-electricity conversion. While this value represents an idealized upper estimate, it serves to illustrate the significant resource potential of WPPG when considered as a sustainable feedstock for large-scale energy harvesting. This is comparable to the output of multiple nuclear power plants and suggests the feasibility of the use of WPPG as a decentralized energy source for low-power electronics that provides a scalable solution for large-scale sustainable energy production.

## Data Availability

The original contributions presented in this study are included in the article/Supplementary Material. Further inquiries can be directed to the corresponding author.

## Supplementary Materials

The following supporting information can be downloaded at: https://www.mdpi.com/article/10.3390/polym17212945/s1, Figure S1: Experimental setup and settings for temperature and humidity control used in the measurements of the current, voltage and resistance of WPPG; Figure S2: SEM image and EDS elemental mapping of WPPG. The SEM image shows the surface morphology of the WPPG, while the corresponding EDS mapping confirms the uniform distribution of carbon, oxygen, and other elements on the paper matrix after carbon black coating.; Figure S3: Raw data used for the evaluation of *I*_sc_ and *V*_oc_ of WPPGs. (a) *I*_sc_ for WPPG-stack. (b) *V*_oc_ for WPPG-stack. (c) *I*_sc_ for WPPG-shred. (d) *V*_oc_ for WPPG-shred.; Figure S4: Raw data for the evaluation of *I*_sc_ and *V*_oc_ of WPPG-shred devices fabricated with different carbon black concentrations. (a) *I*_sc_ for 3.75 g/mL. (b) *V*_oc_ for 3.75 g/mL. (c) *I*_sc_ for 6.25 g/mL. (d) *V*_oc_ for 6.25 g/mL. (e) *I*_sc_ for 8.75 g/mL. (f) Voc for 8.75 g/mL. (g) *I*_sc_ for 11.25 g/mL. (h) *V*_oc_ for 11.25 g/mL. (i) *I*_sc_ for 13.75 g/mL. (j) *V*_oc_ for 13.75 g/mL.; Figure S5: Zeta potential of carbon black (CB) dispersion solutions as a function of CB concentration.; Figure S6: Transmission electron microscopy (TEM) images of carbon black particles.; Figure S7: Resistance of the WPPG-shred devices as a function of carbon black concentration.; Figure S8: Raw data for the evaluation of *I*_sc_ and *V*_oc_ of WPPG-shred device fabricated using different masses of wasted printing paper. (a) *I*_sc_ for 0.5 g. (b) *V*_oc_ for 0.5 g. (c) *I*_sc_ for 1.0 g. (d) *V*_oc_ for 1.0 g. (e) *I*_sc_ for 1.5 g. (f) *V*_oc_ for 1.5 g. (g) *I*_sc_ for 2.0 g. (h) *V*_oc_ for 2.0 g.; Figure S9: Resistance of WPPG-shred devices as a function of mass of wasted printing paper.; Figure S10: WPPG-shred thickness and density versus WPP mass.; Figure S11: WPPG-shred *V*_oc_ and *I*_sc_ for different injection solutions.; Figure S12: *V*_oc_, *I*_sc_ and resistance for WPPG-shred devices with different generator widths (10 and 20 mm) and a length of 20 mm.; Figure S13: *V*_oc_, *I*_sc_ and resistance of WPPG-shred devices with different lengths (10, 20, 30, and 40 mm) and a width of 20 mm.; Figure S14: Raw for the evaluation of *I*_sc_ and *V*_oc_ of WPPG-shred with different injection solutions. (a) *I*_sc_ for deionized water (300 μL). (b) *V*_oc_ for deionized water (300 μL). (c) *I*_sc_ 3M CaCl2 (300 μL). (d) *V*_oc_ for 3M CaCl2 (300 μL).; Figure S15: Raw data for the evaluation of *I*_sc_ and *V*_oc_ of WPPG-shred devices with different generator width (20 and 40 mm). (a) *I*_sc_ for 20 mm. (b) *V*_oc_ for 20 mm. (c) *I*_sc_ for 40 mm. (d) *V*_oc_ for 40 mm.; Figure S16: Raw data for the evaluation *I*_sc_ and *V*_oc_ of WPPG-shred generators with different lengths (10, 20, 30 and 40 mm). (a) *I*_sc_ for 10 mm. (b) *V*_oc_ for 10 mm. (c) *I*_sc_ for 20 mm. (d) *V*_oc_ for 20 mm. (e) *I*_sc_ for 30 mm. (f) *V*_oc_ for 30 mm. (g) *I*_sc_ for 40 mm. (h) *V*_oc_ for 40 mm.; Table S1: Two structural configurations of the WPP generator used in this study—WPPG-stack (25 sheet of paper stacked) and WPPG-shred (shredded printing paper).; Table S2: Average weight variation of WPPG-stack and WPPG-shred before and after water absorption. The data represent the average weight change after immersion, based on multiple measurements excluding the maximum and minimum values.
