# Supplementary material for: Hydroelectricity Generation from Fiber-Oriented Waste Paper via Capillary-Driven Charge Separation
Source: Polymers (Basel). 2025 Nov 4;17(21):2945. doi: 10.3390/polym17212945 (PMC12608816; doi:10.3390/polym17212945)
Supplement: Supplementary file 1 [file polymers-17-02945-s001.zip › polymers-3930121-supplementary.pdf]

Supplementary Information

Hydroelectricity Generation from Fiber-Oriented Waste Paper via Capillary-Driven Charge Separation

Hyun-Woo Lee[1, 2], Seung-Hwan Lee[1], So Hyun Baek[1], Yongbum Kwon[1], Mi Hye Lee[1], Kanghyuk Lee[1], Inhee Cho[1], Bum Sung Kim[1], Haejin Hwang[2], Da-Woon Jeong[1, 3, *]

[1] Korea National Institute of Rare Metals, Korea Institute of Industrial Technology, Incheon 21655, Republic of Korea

[2] Department of Material Science Engineering, Inha University, Incheon 22212, Republic of Korea

[3] School of Mechanical Engineering, Chung-Ang University, Seoul 06974, Republic of Korea

* Corresponding author: Tel: +82-32-226-1362; Fax: +82-32-226-1374;
E-mail: dwjeong@kitech.re.kr

Table S1. Two structural configurations of the WPP generator used in this study—WPPG-stack (25 sheet of paper stacked) and WPPG-shred (shredded printing paper)

Structure type	Description	Sample Size	Thickness	Weight
WPPG–stack	25 sheets stacked and molded	20 × 40 mm	1.8 mm	1.5 g
WPPG–shred	Shredded printing paper mixed with CB solution, molded	20 × 40 mm	1.9 mm	1.5 g

Table S2. Average weight variation of WPPG-stack and WPPG-shred before and after water absorption. The data represent the average weight change after immersion, based on multiple measurements excluding the maximum and minimum values.

Shred type	Weight Before Water Absorption (g)	Weight After Water Absorption (g)	Weight Variation (g)	Average Weight Variation (Excluding Min/Max) (g)
1	1.5029	2.3443	0.8414	
2	1.4960	2.0333	0.5373	
3	1.5091	1.9016	0.3925	0.5904
4	1.4977	1.8657	0.3680	
5	1.5019	2.7157	1.2138	
Stack type	Weight Before Water Absorption (g)	Weight After Water Absorption (g)	Weight Variation (g)	Average Weight Variation (Excluding Min/Max) (g)
1	1.5448	1.6872	0.1424	
2	1.5893	1.9047	0.3154	
3	1.5427	1.6333	0.0906	0.2442
4	1.5443	1.8382	0.2939	
5	1.5131	1.8096	0.2965	

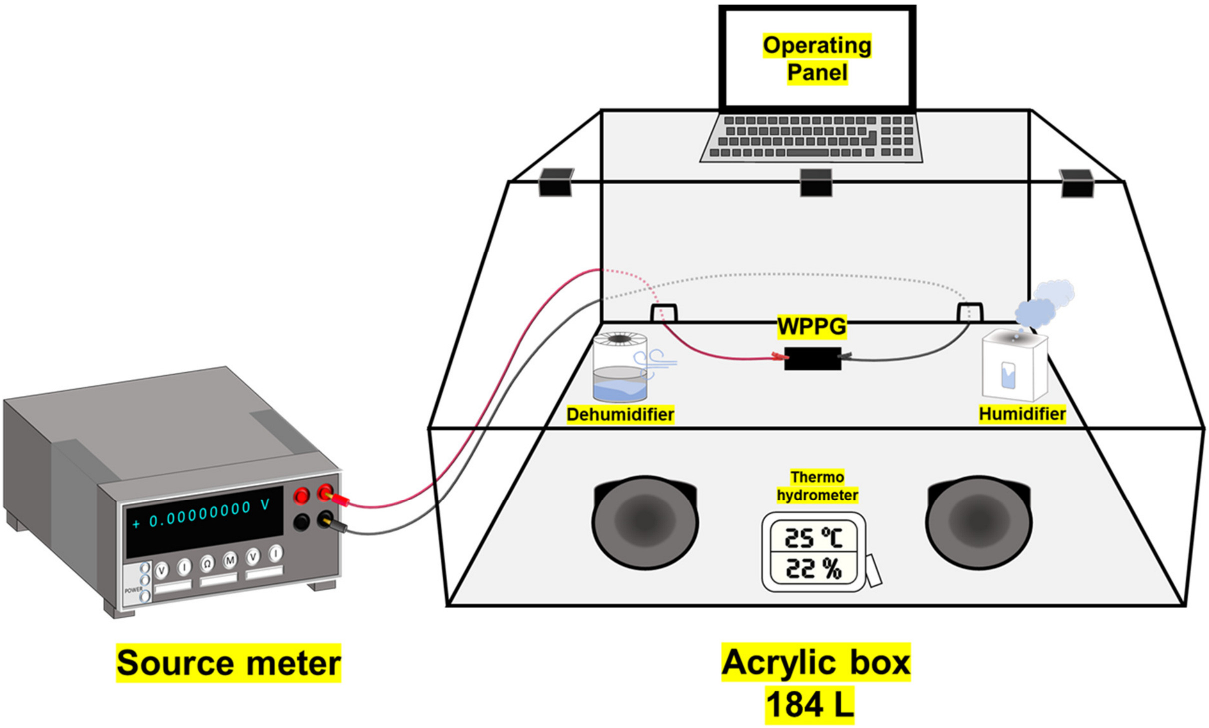

Figure S1. Experimental setup and settings for temperature and humidity control used in the measurements of the current, voltage and resistance of WPPG.

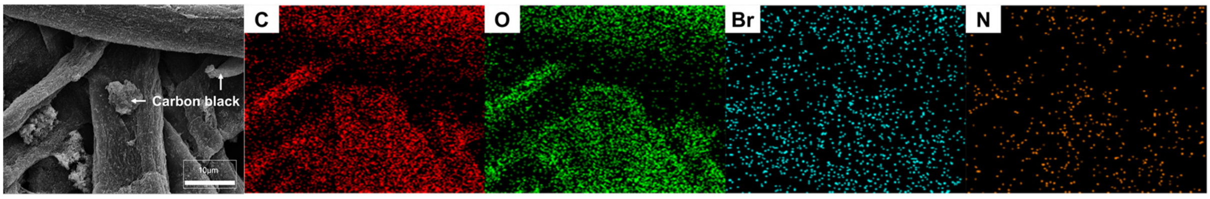

Figure S2. SEM image and EDS elemental mapping of WPPG. The SEM image shows the surface morphology of the WPPG, while the corresponding EDS mapping confirms the uniform distribution of carbon, oxygen, and other elements on the paper matrix after carbon black coating.

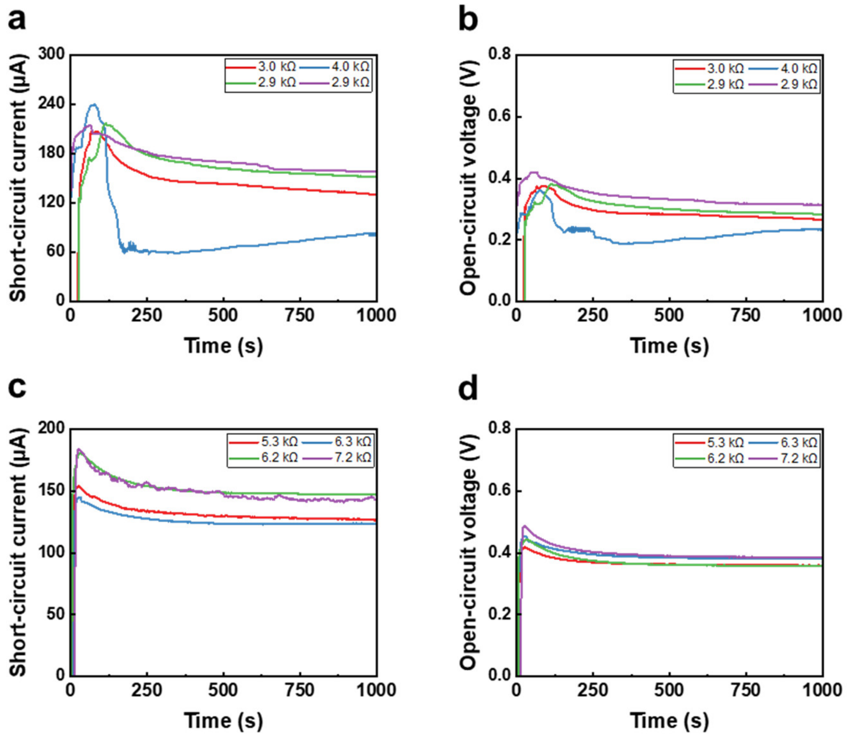

Figure S3. Raw data used for the evaluation of I_{sc} and V_{oc} of WPPGs. a) I_{sc} for WPPG-stack. b) V_{oc} for WPPG-stack. c) I_{sc} for WPPG-shred. d) V_{oc} for WPPG-shred.

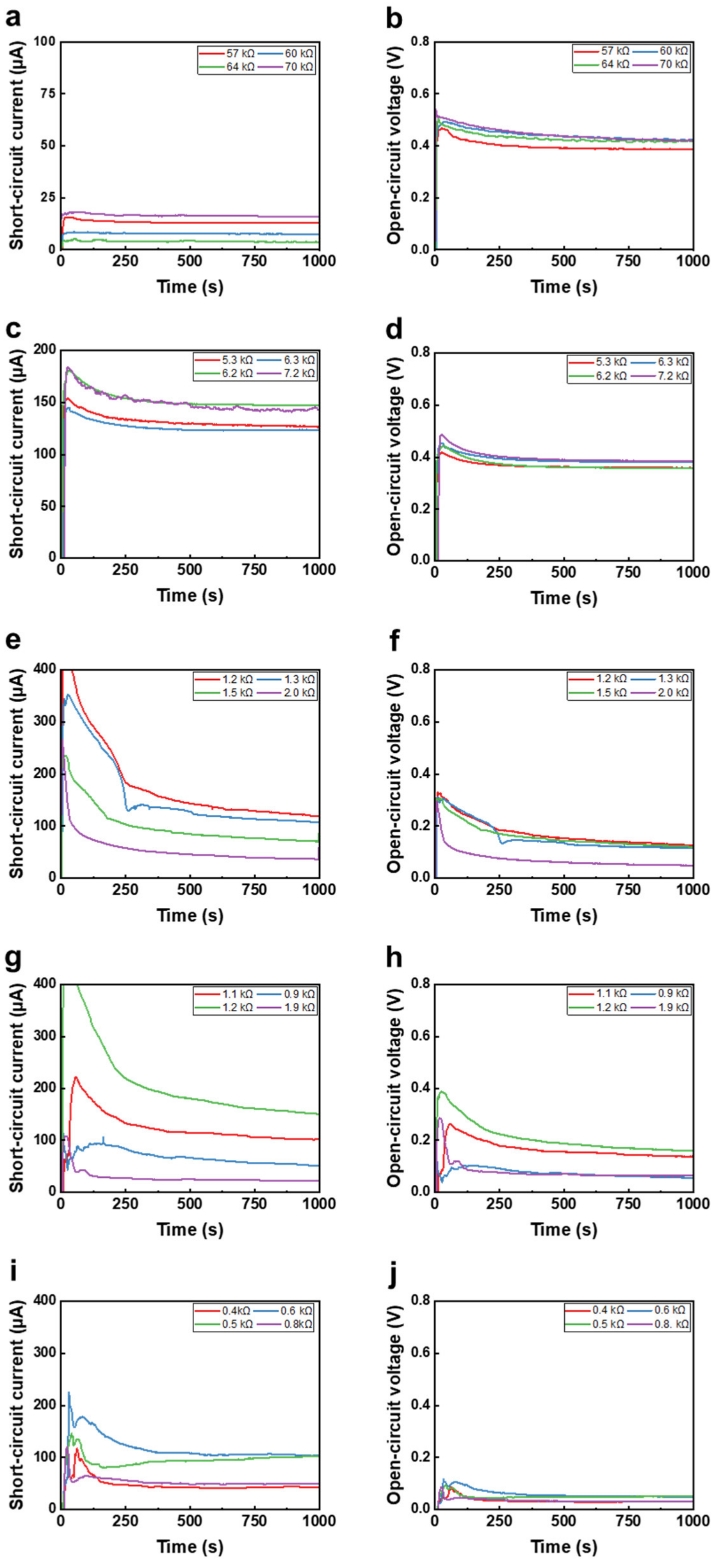

Figure S4. Raw data for the evaluation of I_{sc} and V_{oc} of WPPG-shred devices fabricated with different carbon black concentrations. a) I_{sc} for 3.75 g/mL. b) V_{oc} for 3.75 g/mL. c) I_{sc} for 6.25 g/mL. d) V_{oc} for 6.25 g/mL. e) I_{sc} for 8.75 g/mL. f) V_{oc} for 8.75g/mL. g) I_{sc} for 11.25 g/mL. h) V_{oc} for 11.25g/mL. i) I_{sc} for 13.75 g/mL. j) V_{oc} for 13.75 g/mL.

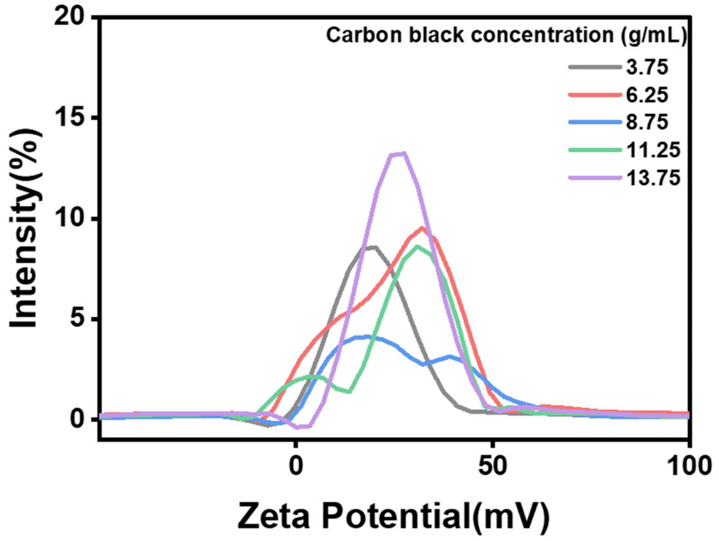

Figure S5. Zeta potential of carbon black (CB) dispersion solutions as a function of CB concentration.

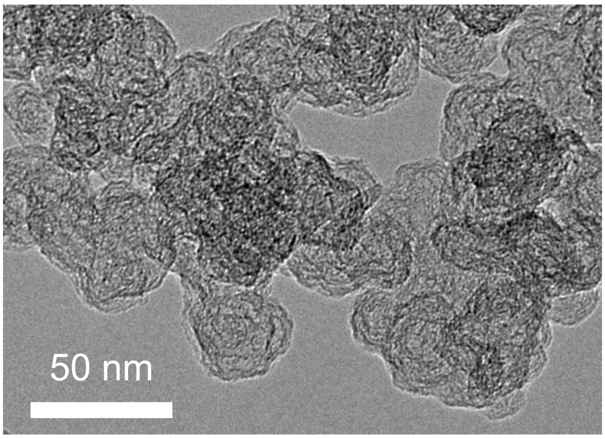

Figure S6. Transmission electron microscopy (TEM) images of carbon black particles.

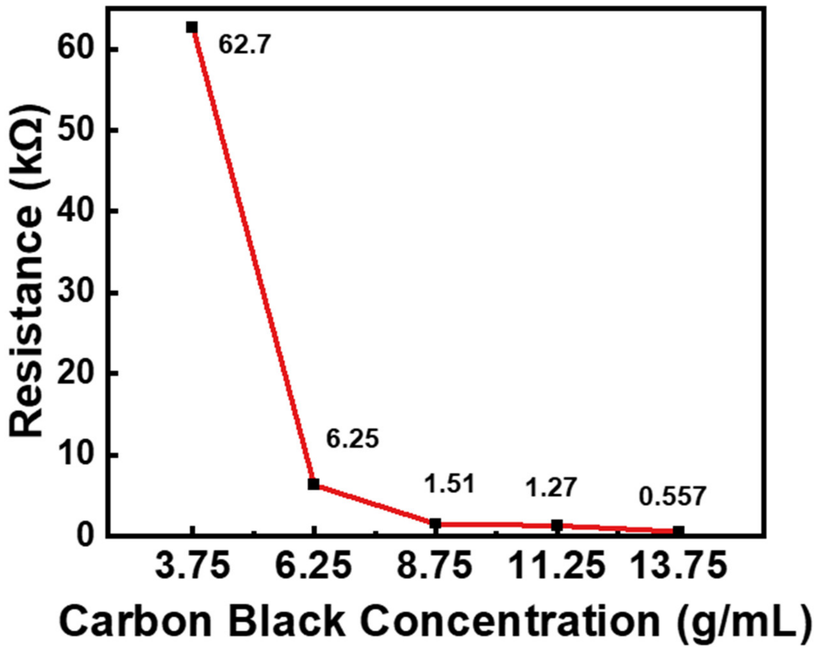

Figure S7. Resistance of the WPPG-shred devices as a function of carbon black concentration.

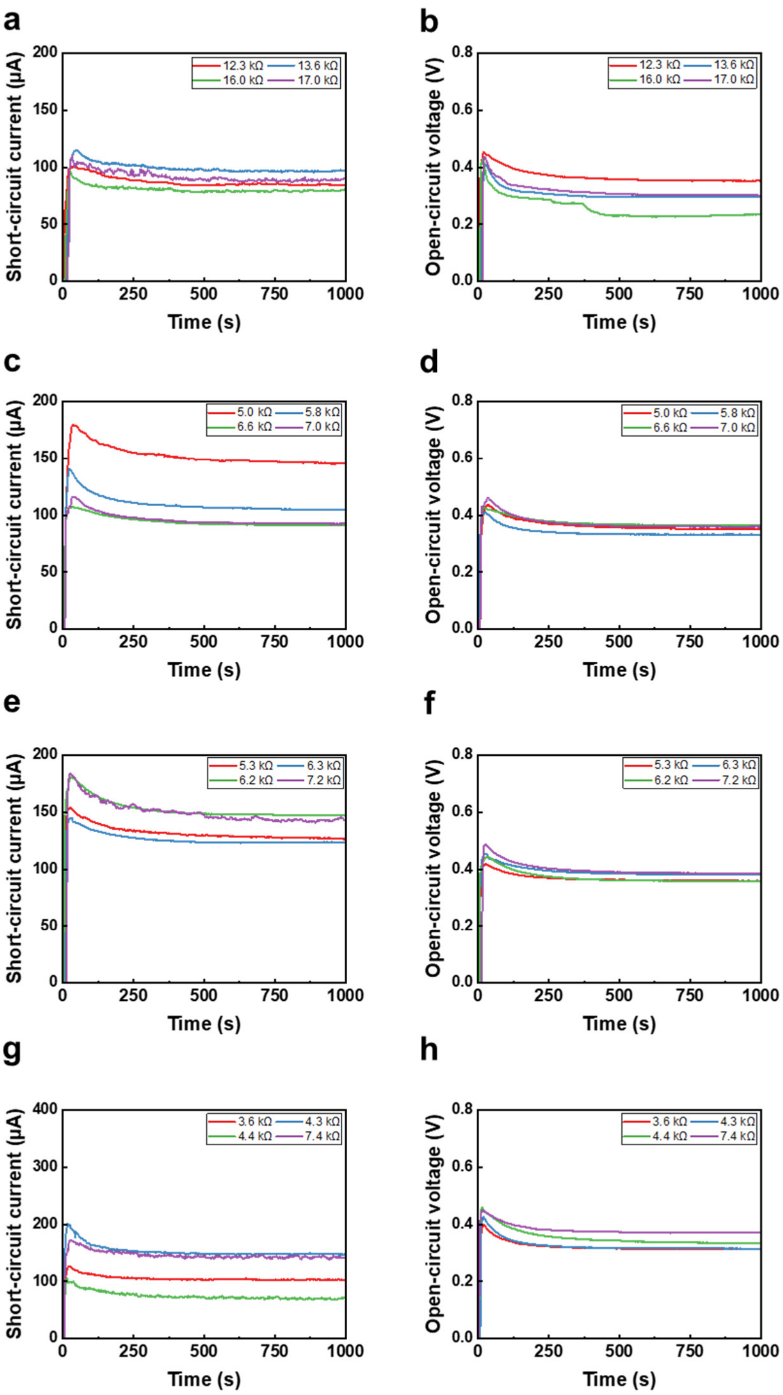

Figure S8. Raw data for the evaluation of I_{sc} and V_{oc} of WPPG-shred device fabricated using different masses of wasted printing paper. a) I_{sc} for 0.5 g. b) V_{oc} for 0.5 g. c) I_{sc} for 1.0 g. d) V_{oc} for 1.0 g. e) I_{sc} for 1.5 g. f) V_{oc} for 1.5 g. g) I_{sc} for 2.0 g. h) V_{oc} for 2.0 g.

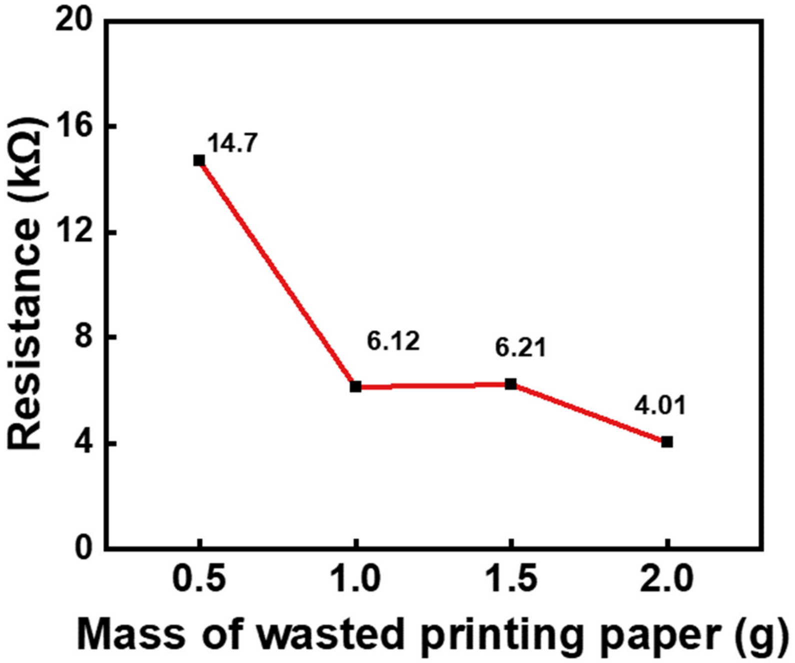

Figure S9. Resistance of WPPG-shred devices as a function of mass of wasted printing paper.

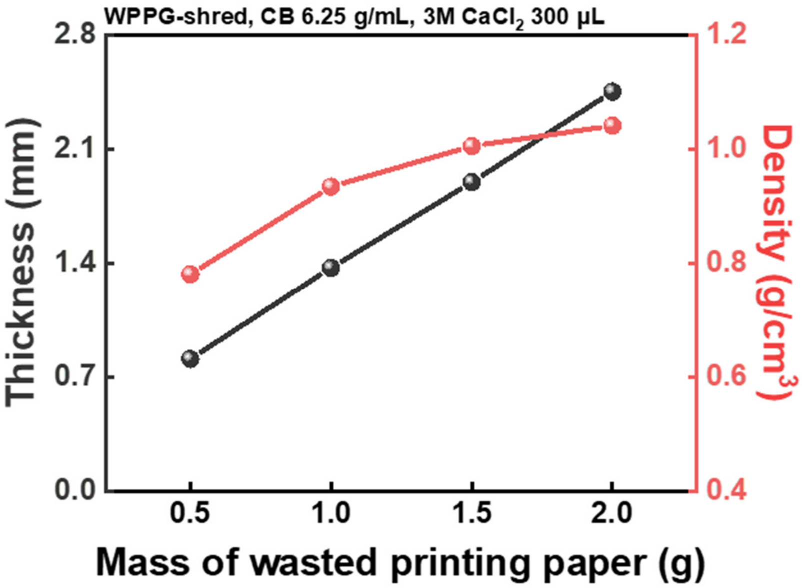

Figure S10. WPPG-shred thickness and density versus WPP mass

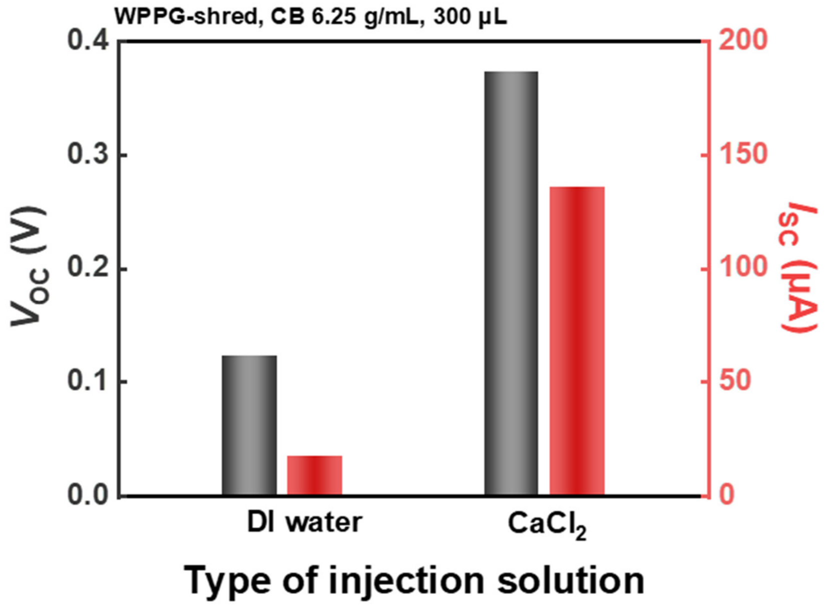

Figure S11. WPPG-shred V_{oc} and I_{sc} for different injection solutions

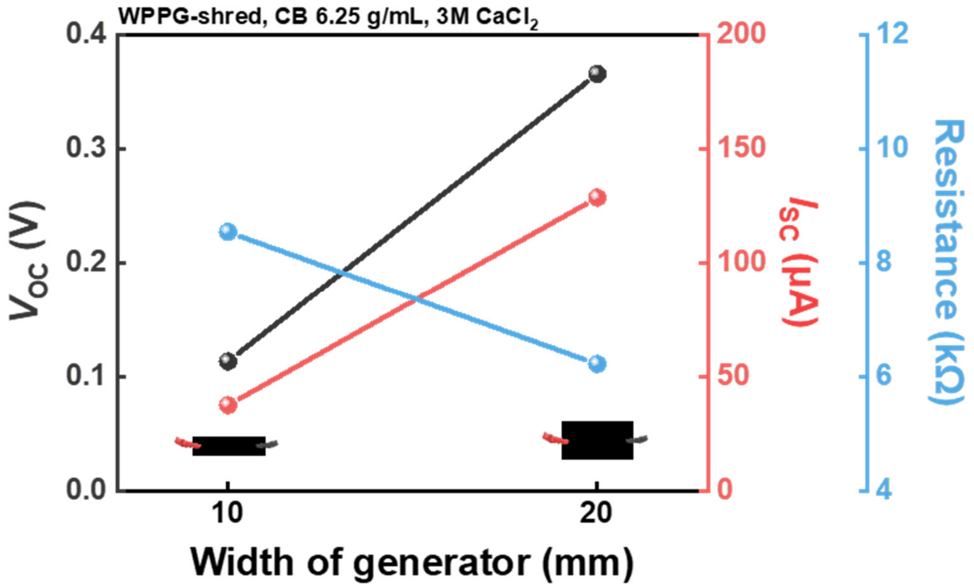

Figure S12. V_{oc}, I_{sc} and resistance for WPPG-shred devices with different generator widths (10 and 20 mm) and a length of 20 mm

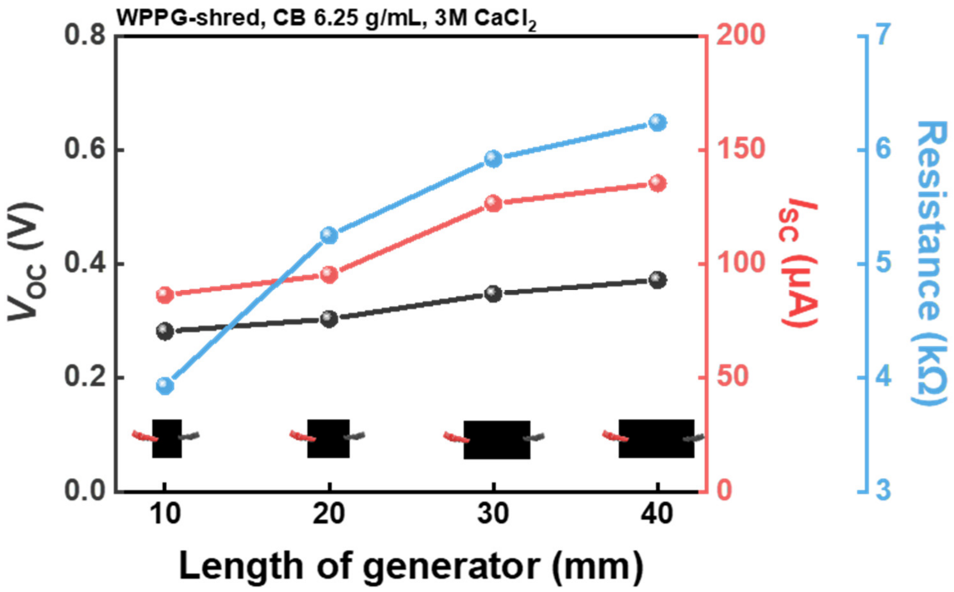

Figure S13. V_{oc}, I_{sc} and resistance of WPPG-shred devices with different lengths (10, 20, 30, and 40 mm) and a width of 20 mm

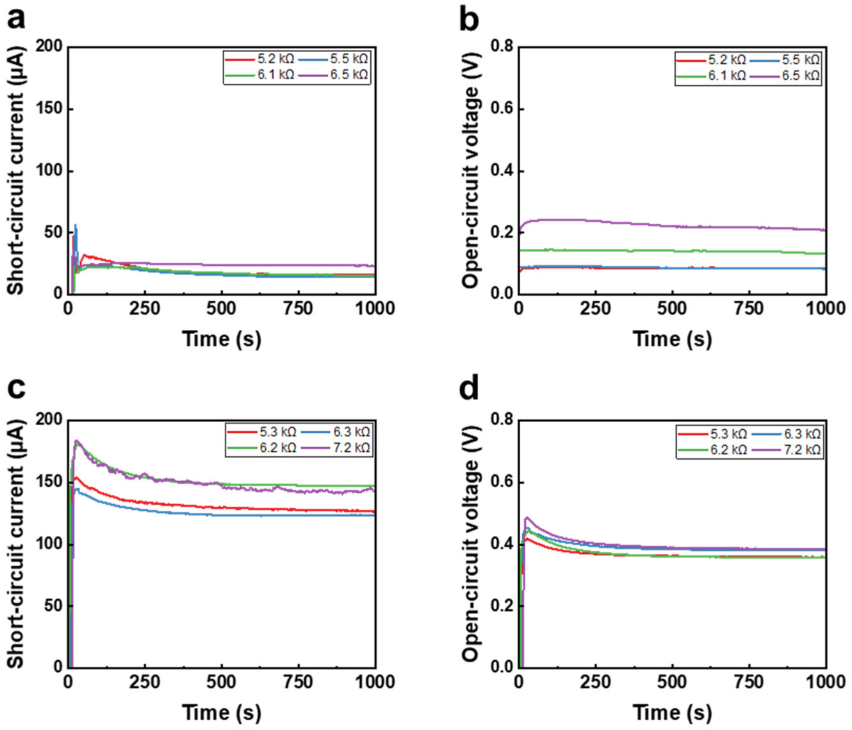

Figure S14. Raw for the evaluation of I_{sc} and V_{oc} of WPPG-shred with different injection solutions. a) I_{sc} for deionized water (300 μL). b) V_{oc} for deionized water (300 μL). c) I_{sc} 3M $CaCl_2$ (300 μL). d) V_{oc} for 3M $CaCl_2$ (300 μL).

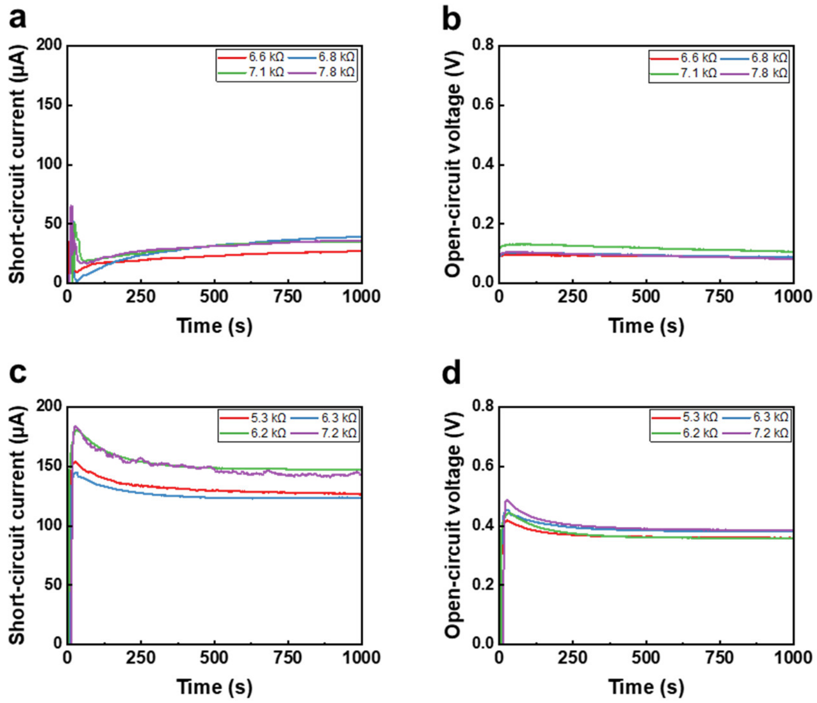

Figure S15. Raw data for the evaluation of I_{sc} and V_{oc} of WPPG-shred devices with different generator width (20 and 40 mm). a) I_{sc} for 20 mm. b) V_{oc} for 20 mm. c) I_{sc} for 40 mm. d) V_{oc} for 40 mm.

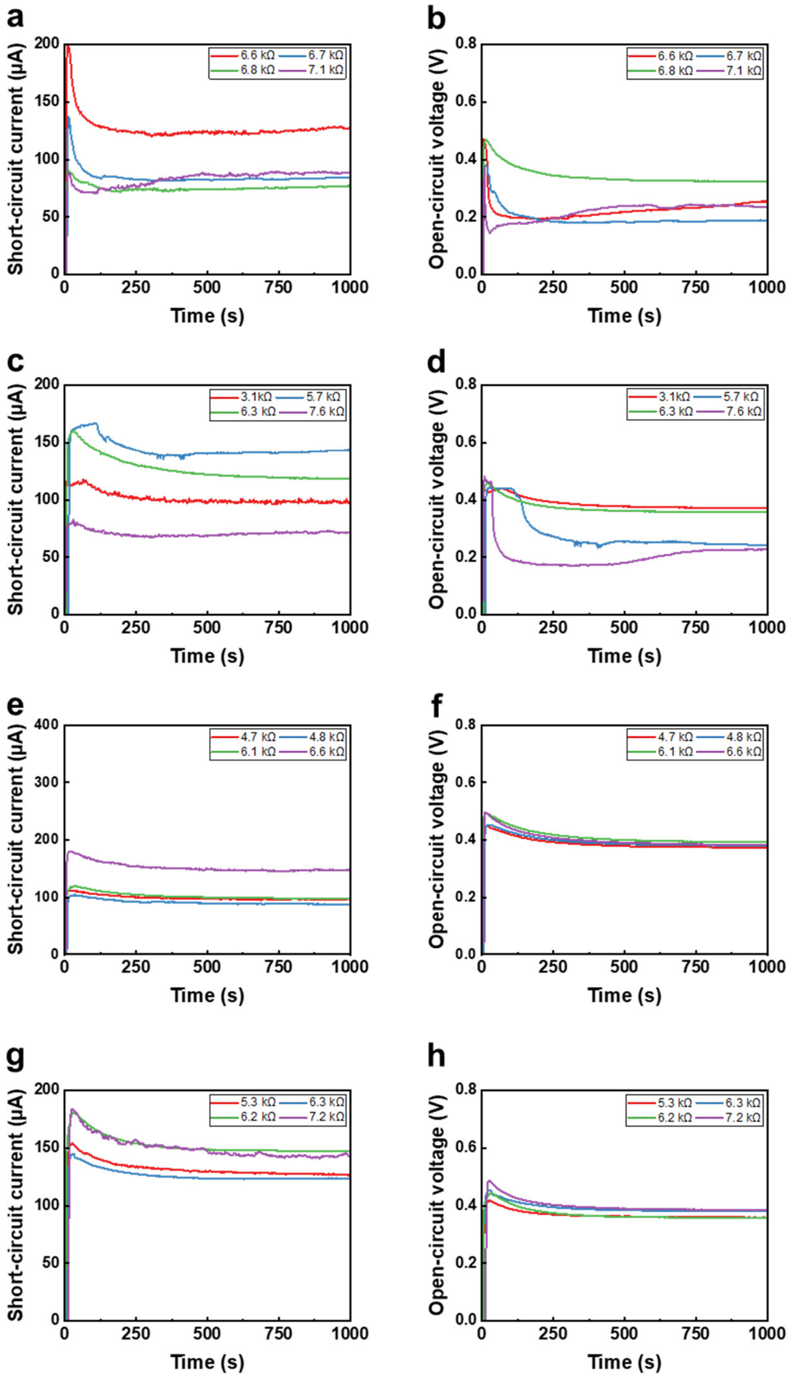

Figure S16. Raw data for the evaluation I_{sc} and V_{oc} of WPPG-shred generators with different lengths (10, 20, 30 and 40 mm). a) I_{sc} for 10 mm. b) V_{oc} for 10 mm. c) I_{sc} for 20 mm. d) V_{oc} for 20 mm. e) I_{sc} for 30 mm. f) V_{oc} for 30 mm. g) I_{sc} for 40 mm. h) V_{oc} for 40 mm.
